# SeQuiLa-cov: A fast and scalable library for depth of coverage calculations

**DOI:** 10.1093/gigascience/giz094

**Published:** 2019-08-05

**Authors:** Marek Wiewiórka, Agnieszka Szmurło, Wiktor Kuśmirek, Tomasz Gambin

**Affiliations:** Institute of Computer Science, Warsaw University of Technology, ul. Nowowiejska 15/19, 00-665 Warsaw, Poland

**Keywords:** NGS data analysis, depth of coverage, big data, distributed computing, SQL, CNV-calling, RNA-seq, quality control for sequencing data

## Abstract

**Background:**

Depth of coverage calculation is an important and computationally intensive preprocessing step in a variety of next-generation sequencing pipelines, including the analysis of RNA-sequencing data, detection of copy number variants, or quality control procedures.

**Results:**

Building upon big data technologies, we have developed SeQuiLa-cov, an extension to the recently released SeQuiLa platform, which provides efficient depth of coverage calculations, reaching >100× speedup over the state-of-the-art tools. The performance and scalability of our solution allow for exome and genome-wide calculations running locally or on a cluster while hiding the complexity of the distributed computing with Structured Query Language Application Programming Interface.

**Conclusions:**

SeQuiLa-cov provides significant performance gain in depth of coverage calculations streamlining the widely used bioinformatic processing pipelines.

## Findings

 

## Introduction

Given a set of sequencing reads and a genomic contig, depth of coverage for a given position is defined as the total number of reads overlapping the locus.

The coverage calculation is a frequently performed but time-consuming step in the analysis of next-generation sequencing (NGS) data. In particular, copy number variant detection pipelines require obtaining sufficient read depth of the analyzed samples [1–3]. In other applications, the coverage is computed to assess the quality of the sequencing data (e.g., to calculate the percentage of genome with ≥30× read depth) or to identify genomic regions overlapped by an insufficient number of reads for reliable variant calling [4]. Finally, depth of coverage is one of the most computationally intensive parts of differential expression analysis using RNA-sequencing data at single-base resolution [5–7].

A number of tools supporting this operation have been developed, with 22 of them specified in the Omictools catalog [8]. Well-known, state-of-the-art solutions include samtools depth [9], bedtools genomecov [10], GATK DepthOfCoverage [11], sambamba [12], and mosdepth [13] (see comparison presented in Table 1).

**Table 1:** Comparison of leading coverage calculation software tools

		Functionality				Implementation		
Tool	Approach	Bases	Blocks	Windows	Language	Intel GKL	Parallelism type	Interface
**samtools**	Pileup	Yes	No	No	C	No	None	Command line
**bedtools**	Events	Yes	Yes	No	C++	No	None	Command line
**GATK** ^1^	Pileup	Yes	No	No	Java	Yes	Distributed	Command line
**sambamba**	Pileup	No	Yes	Yes	D	No	Multithreaded	Command line
**mosdepth**	Events	No	Yes	Yes	Nim	No	Multithreaded^2^	Command line
SeQuiLa-cov	Events	Yes	Yes	Yes	Scala	Yes	Distributed	Scala, SQL

1 GATK DepthOfCoverage has not yet been ported to the latest version, i.e., GATK 4.x.

2 Only for BAM decompression.

**Figure 1:**
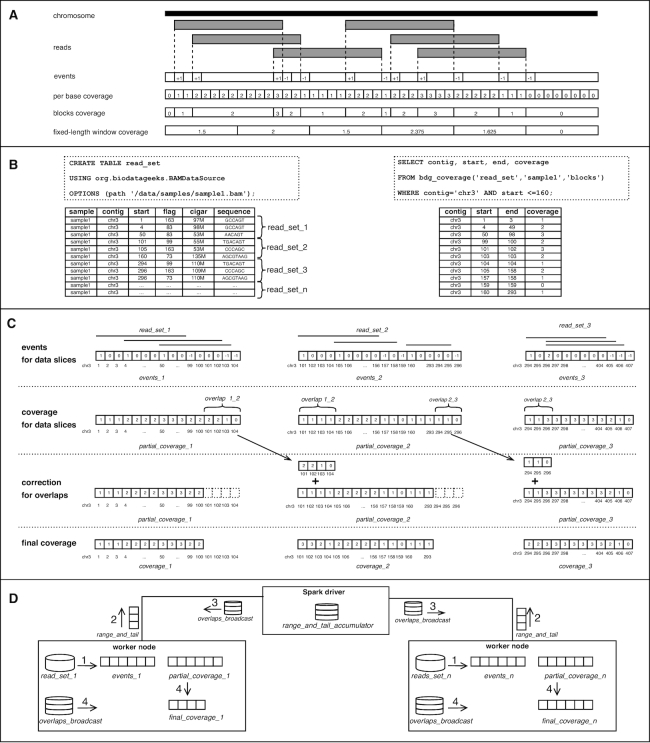
SeQuiLa-cov: functionality, algorithm, and implementation. **(A)** General concept of events-based algorithm for depth of coverage calculation. Given a genomic chromosome and a set of aligned sequencing reads, the algorithm allocates “events” vector. Subsequently, it iterates the list of reads and increments/decrements by 1 the values of the events vector at the indexes corresponding to start/end positions of each read. The depth of coverage for a genomic locus is calculated using the cumulative sum of all elements in the events vector preceding the specified position. The algorithm may produce 3 typically used coverage types: (i) per-base coverage, which includes the coverage value for each genomic position separately, (ii) blocks, which lists adjacent positions with equal coverage values merged into a single interval, and (iii) fixed-length windows coverage, which generates a set of equal-size, non-overlapping and tiling genomic ranges and outputs the arithmetic mean of base coverage values for each region. **(B)** Provided SQL API to interact with NGS data. The first statement creates a relational table read_set over compressed BAM files using the provided custom Data Source, whereas the second statement demonstrates the use of the bdg_coverage function to calculate depth of coverage for a specified sample. The presented call for coverage method takes sample identifier (sample1) and result type (blocks) as input parameters. bdg_coverage is implemented as a table-valued function. Therefore, it outputs a table as a result, allowing for customizing a query using Data Manipulation Language, e.g., in the SELECT or WHERE clause. For the purpose of this example, we assume that the BAM file for sample1 contains only reads from chr3. **(C)** Concept of distributed version of events-based algorithm. Assuming that we run our calculations in a distributed environment, the computation nodes do not work on the whole input data set (table read_set) but on *n* smaller data partitions (slice_1_, slice_2_, ..., slice_*n*_), each containing a subset of input aligned reads. The algorithm first calculates the partial events vector for available data slices and subsequently produces a corresponding partial partial_coverage vector. Because of the possibility of overlapping of ranges between 2 consecutive data slices, an additional correction step needs to be performed. When an overlap is identified, the corresponding coverage values from the preceding vector’s tail are cut and added to the head values of the subsequent vector. On the figure, 2 overlaps are shown, one of them situated between partial_coverage_1_ and partial_coverage_2_ (overlap_12_ of length 4) encompassing positions chr3:101–104. The coverage values from partial_coverage_1_ for overlap_12_ are removed from partial_coverage_1_ and added to the head of partial_coverage_2_. As a result, a set of non-overlapping coverage vectors are calculated, which is further integrated into the depth of coverage for the whole input data set. **(D**) Implementation details of SeQuiLa-cov. We have used the Apache Spark environment, where a single driver node runs the high-level driver program, which schedules tasks for multiple worker nodes. On each worker node, a set of data partitions are accessed and manipulated in order to generate events and partial_coverage vectors. To gather data about partial_coverage vectors’ ranges along with tailing coverage values, and to distribute data needed for rearranging coverage vector values and ranges, we have used Spark’s shared variables “accumulator” and “broadcast,” respectively.

Traditionally, these methods calculate the depth of coverage using a pileup-based approach (introduced in samtools [9] and used in GATK [11]), which is inefficient because it iterates through each nucleotide position at every read in a BAM file. An optimized, event-bas^10^] and mosdepth [13]. These algorithms use only specific “events,” i.e., start and end of the alignment blocks within each read (Fig. 1A) instead of analyzing every base of each read, which substantially reduces the overall computational complexity.

Samtools and bedtools depth of coverage modules do not provide any support for a multi-core environment. Mosdepth implements parallel BAM decompression, but its main algorithm remains sequential. Sambamba, on the other hand, promotes itself as a highly parallel tool, implementing depth of coverage calculations in a map-reduce fashion using multiple threads on a single node. Regardless of parallelization degree, all of the aforementioned tools share a common bottleneck caused by using a single thread for returning results. Finally, GATK was the first genomic framework to provide support for distributed computations; however, the DepthOfCoverage method has not yet been ported to the current software release of the toolkit.

We present the first fully scalable, distributed, SQL-oriented solution designated for depth of coverage calculations. SeQuiLa-cov, an extension to the recently released SeQuiLa [14] platform, runs a redesigned event-based algorithm for the distributed environment and provides a convenient, SQL-compliant interface.

## Algorithm and implementation

### Algorithm

Consider an input data set, read_set, of aligned sequencing reads sorted by genomic position from a BAM file partitioned into *n* data slices (read_set_1_, read_set_2_, read_set*_n_*) (Fig. 1B).

In the most general case, the algorithm can be used in a distributed environment where each cluster node computes the coverage for the subset of data slices using the event-based method. Specifically, for the *i*th partition containing the set of reads (read_set*_i_*), the set of events_*i*,chr_ vectors (where chr is an index of genomic contig represented in read_set) is allocated and updated, based on the items from read_set*_i_*. For all reads, the algorithm parses the concise idiosyncratic gapped alignment report (CIGAR) string, and for each continuous alignment block characterized by start position and length len it increments by 1 the events_*i,chr*_(start) and decrements by 1 the value of events_*i,chr*_(start + len). To compute the partial coverage vector for partition *i* and contig chr, a vector value at the index *j* is calculated as follows:

\begin{equation*}
\mathrm{partial}\_\mathrm{coverage}_{i,\mathrm{chr}}(j) = \sum \nolimits _{m=1}^{j} \mathrm{events}_{i,\mathrm{chr}}(m).
\end{equation*}

The result of this stage is a set of partial_coverage_*i*,chr_ vectors distributed among the computation nodes. To calculate the final coverage for the whole read_set, an additional step of correction for overlaps between the partitions is required. An overlap overlap_*i*,chr_ of length *l* between vectors partial_coverage_*i*,chr_ and partial_coverage_*i*+1,chr_ may occur on the partition boundaries where *l* tailing genomic positions of partial_coverage_*i*,chr_ are the same as *l* heading genomic positions of partial_coverage_*i*+1,chr_ (see Fig. 1C).

If an overlap is identified, then the coverage values from the partial_coverage_*i*,chr_’s *l*-length tail are added into the partial_coverage_*i*+1,chr_’s head and subsequently the last *l* elements of partial_coverage_*i*,chr_ are removed. Once this correction step is completed, non-overlapping coverage_*i*,chr_ vectors are collected and yield the final coverage values for the whole input read_set.

The main characteristic of the described algorithm is its ability to distribute data and calculations (such as BAM decompression and main coverage procedure) among the available computation nodes. Moreover, instead of simply performing the full data reduction stage of the partial coverage vectors, our solution minimizes required data shuffling among cluster nodes by limiting it to the overlapping part of coverage vectors. Importantly, the SeQuiLa-cov computation model supports fine-grained parallelism at a user-defined partition size in contrast to the traditional, coarse-grained parallelization strategies that involve splitting input data at a contig level.

### Implementation

We have implemented SeQuiLa-cov in Scala programming language using the Apache Spark framework. To efficiently access the data from a BAM file we have prepared a custom data source using Data Source API exposed by SparkSQL. Performance of the read operation benefits from the Intel Genomics Kernel Library (GKL) [15] used for decompressing the BAM file chunks and from a predicate push-down mechanism that filters out data at the earliest stage.

The implementation of the core coverage calculation algorithm aimed to minimize the memory footprint whenever possible by using parsimonious data types, e.g., “Short” type instead of “Integer,” and to implement an efficient memory allocation strategy for large data structures, e.g., favoring static Arrays over dynamic size ArrayBuffers. Additionally, to reduce the overhead of data shuffling between the worker nodes in the correction for overlap stage, we used Spark’s shared variables [16] “accumulators” and “broadcast variables” (Fig. 1C). Accumulator is used to gather information about the worker nodes’ coverage vector ranges and coverage vector tail values, which are subsequently read and processed by the driver. This information is then used to construct a broadcast variable distributed to the worker nodes in order to perform adequate trimming and summing operations on partial coverage vectors.

## Functionality

### Supported coverage result types

SeQuiLa-cov features 3 distinct result types: “per-base,” “blocks,” and “fixed-length windows” coverage (Fig. 1A). For per-base, the depth of coverage is calculated and returned for each genomic position, making it the most verbose output option. The method producing block-level coverage (blocks) involves merging adjacent genomic positions with equal coverage values into genomic intervals. As a consequence, fewer records than in the case of per-base output type are generated, with no information loss. For the fixed-length windows the algorithm generates set of fixed-length, tiling, non-overlapping genomic intervals and returns the arithmetic mean of coverage values over positions within each window.

### ANSI SQL compliance

The SeQuiLa-cov solution promotes SQL as a data query and manipulation language in genomic analysis. Data flows are performed in SQL-like manner through the custom data source, supporting the convenient Create Table as Select and Insert as Select methods. SeQuiLa-cov provides a table abstraction over existing alignment files, with no need of data conversion, which can be further queried and manipulated in a declarative way. The coverage calculation function bdg_coverage, as described in the Algorithm subsection, has been implemented as a table-valued function (Fig. 1D).

### Execution and integration options

SeQuiLa-cov can be used as an extension to Apache Spark in the form of an external JAR dependency or can be executed from the command line as a Docker container. Both options can be run locally (on a single node) or on a Hadoop cluster using YARN (see project documentation for sample commands). The tool accepts BAM/CRAM files as input and supports processing of short and long reads. The tabular output of the coverage computations can be stored in various file formats, e.g., binary (ORC, Parquet), as well as text (CSV, TSV). The tool can be integrated with state-of-the-art applications through text files or can be used directly as an additional library in bioinformatics pipelines implemented in Scala, R, or Python.

## Benchmarking

We have benchmarked SeQuiLa-cov solutions with leading software for depth of coverage calculations, specifically samtools depth, bedtools genomeCov, sambamba depth, and mosdepth (results of DepthOfCOverage from outdated GATK version are available at http://biodatageeks.org/sequila/benchmarking/benchmarking.html#depth-of-coverage). The tests were performed on the aligned whole-exome sequencing (WES) and whole-genome sequencing (WGS) reads from the NA12878 sample (see Methods for details) and aimed at calculating blocks and window coverage. To compare the performance and scalability of each solution, we executed calculations for 1, 5, and 10 cores on a single computation node (see Table 2).

**Table 2:** Benchmarking leading solutions against SeQuiLa-cov on WES/WGS data in execution time of block and window calculations

Data	Operation type	Cores	samtools	bedtools	sambamba	mosdepth	SeQuiLa-cov
WGS	Blocks	1	2h 14m 58s^1^	10h 41m 27s	2h 44m 0s	1h 46m 27s	1h 47m 5s
		5			2h 47m 53s	36m 13s	26m 59s
		10			2h 50m 47s	34m 34s	13m 54s
	Fixed-length windows	1			1h 46m 50s	1h 22m 49s	1h 24m 8s
		5			1h 41m 23s	20m 3s	18m 43s
		10			1h 50m 35s	17m 49s	9m 14s
WES	Blocks	1	12m 26s^1^	23m 25s	25m 42s	6m 43s	6m 54s
		5			25m 46s	2m 25s	1m 47s
		10			25m 49s	2m 20s	1m 4s
	Fixed-length windows	1			14m 36s	6m 11s	6m 29s
		5			14m 54s	2m 8s	1m 42s
		10			14m 40s	2m 14s	1m 1s

Both samtools and bedtools calculate coverage using only a single thread; however, their results differ significantly, with samtools being approximately twice as fast. Sambamba positions itself as a multithreaded solution, although our tests revealed that its execution time is nearly constant, regardless of the number of CPU cores used, and even twice as slow as samtools. Mosdepth achieved speedup against samtools in blocks coverage and against sambamba in windows coverage calculations; however, its scalability reaches its limit at 5 CPU cores. Finally, SeQuiLa-cov achieves performance nearly identical to that of mosdepth for the single core, but the execution time decreases substantially for greater number of available computing resources, which makes this solution the fastest when run on multiple cores and nodes.

1 Per-base results are treated as block output. Samtools lacks the functionality of block coverage calculations; however, we included this tool in our benchmark for completeness, treating its per-base results as block outcome assuming that both result types require nearly the same resources.

Samtools depth and bedtools genomeCov are both natively non-scalable and were run on a single thread only. Exome-wide calculations exceeded 10 minutes and genome-wide analyses took >2 hours in the case of samtools, while bedtools’ performance was substantially worse, i.e., ∼1.9× for WES and ∼4.7× for WGS. Sambamba depth claims that it can take advantage of fully parallelized data processing with the use of multithreading. However, our results revealed that even when additional threads were used, the total execution time of coverage calculations remained nearly constant and greater than samtools’ result. Mosdepth shows substantial speedup (∼1.3×) against samtools when using a single thread. This performance gain increases to ∼3.7× when using 5 decompression threads; however, it does not benefit from adding additional CPU power. In the case of fixed-length window coverage mosdepth achieves more than ∼1.3 speedup against sambamba.

SeQuiLa-cov achieves performance similar to mosdepth when run using a single core. However, SeQuiLa-cov is ∼1.3× and ∼2.5× as fast as mosdepth when using 5 and 10 CPU cores, respectively, demonstrating its better scalability. Similar performance is observed for both block and fixed-length window methods.

**Figure 2:**
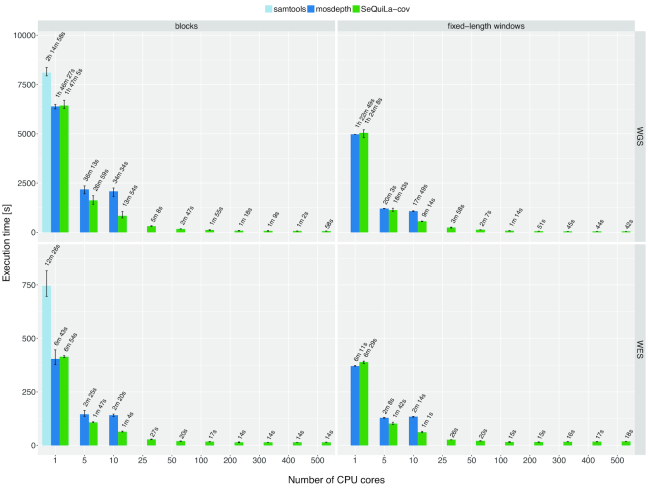
Performance and scalability comparison of samtools, mosdepth, and SeQuiLa-cov. Each experiment setting was repeated several times. Bar height and error bars indicate mean and range of execution time, respectively. The best pileup-based solution is definitely slower (2 times for WGS calculations) than both event-based solutions, which clearly shows the superiority of the latter one. Mosdepth execution time scales up to 5 cores; afterwards it shows no further gain in performance. SeQuiLa-cov has nearly the same execution time results as mosdepth for both block and window calculations for a single core, but scales out desirably using all 500 CPU cores on cluster nodes and at the same time performing WGS calculations in <1 minute.

To fully assess the scalability profile of our solution, we performed additional tests in a cluster environment (see Methods for details). Our results show that when utilizing additional resources (i.e., >10 CPU cores), SeQuiLa-cov is able to reduce the total computation time to 15 seconds for WES and <1 minute for WGS data (Fig. 2). The scalability limit is achieved for 200 and ∼500 CPU cores for WES and WGS data, respectively.

To evaluate the impact of the Intel GKL library on the deflate operation (BAM bzgf block decompression), we performed block coverage calculations on WES data on 50 CPU cores. The results showed on average ∼1.18× speedup when running with the Intel GKL deflate implementation.

Finally, our comprehensive functional unit testing showed that the results calculated by SeQuiLa-cov and samtools depth are identical.

## Conclusions

Recent advances in big data technologies and distributed computing can contribute to speeding up both genomic data processing and management. Analysis of large genomic data sets requires efficient, accurate, and scalable algorithms to perform calculations using the computing power of multiple cluster nodes. In this work, we show that with a sufficiently large cluster, genome-wide coverage calculations may last <1 minute and at the same time be >100× faster than the best single-threaded solution.

Although the tool can be integrated with non-distributed software, our primary aim is to support large-scale processing pipelines, and the full advantage of SeQuiLa-cov’s scalability and performance will be available once it is deployed and executed in a distributed environment. We expect that there will be a growing number of scalable solutions (Big Data Genomics project [17] with tools DECA and Cannoli as well as GATK4 [18]) that can take advantage of reading input data directly from distributed storage systems.

SeQuiLa-cov is one of the building blocks of the SeQuiLa [14] ecosystem, which initiated the move towards efficient, distributed processing of genomic data and providing SQL-oriented API for convenient and elastic querying. We foresee that following this direction will enable the evolution of genomic data analysis from file-oriented to table-oriented processing.

## Methods

### Test data

We tested our solution using reads from the NA12878 sample, which were aligned to the hg18 genome. The WES data contained >161 million reads (17 GB of disk space) and WGS data included >2.6 billion reads (272 GB of disk space). Both BAM files were compressed at the default BAM compression level (5).

### Testing environment

To perform comprehensive performance evaluation, we set up a test cluster consisting of 28 Hadoop nodes (1 edge node, 3 master nodes, and 24 data nodes) with Hortonworks Data Platform 3.0.1 installed. Each data node has 28 cores (56 with hyper-threading) and 512 GB of RAM; YARN resource pool has been configured with 2,640 virtual cores and 9,671 GB RAM.

### Investigated solutions

In our benchmark we used the most recent versions of the investigated tools, i.e., samtools version 1.9, bedtools 2.27.0, sambamba 0.6.8, mosdepth version 0.2.3, and SeQuiLa-cov version 0.5.1.

## Availability of source code and requirements

• Project name: SeQuiLa-cov

• Project home page: http://biodatageeks.org/sequila/

• Source code repository: https://github.com/ZSI-Bio/bdg-sequila

• Operating system: Platform independent

• Programming language: Scala

• Other requirements: Docker

• License: Apache License 2.0

• RRID: SCR_017220

## Availability of supporting data and materials

The Docker image is available at https://hub.docker.com/r/biodatageeks/. Supplementary information on benchmarking procedure as well as test data are publicly accessible at the project documentation site http://biodatageeks.org/sequila/benchmarking/benchmarking.html#depth-of-coverage. An archival copy of the code and supporting data is also available via the GigaScience database GigaDB [19].

## Abbreviations

API: Application Programming Interface; BAM: Binary Alignment Map; CPU: central processing unit; CSV: comma-separated values; GKL: Genomics Kernel Library; NGS: next-generation sequencing; ORC: optimized row columnar; RAM: random access memory; SQL: Structured Query Language; TSV: tab-separated values; YARN: Yet Another Resource Negotiator; WES: whole-exome sequencing; WGS: whole-genome sequencing.

## Competing interests

The authors declare that they have no competing interests.

## Funding

This work has been supported by the Polish budget funds for science in years 2016–2019 (Iuventus Plus grant IP2015 019874), as well as Polish National Science Center grant Preludium 2014/13/N/ST6/01843.

## Authors' contributions

M.W.: conceptualization, formal analysis, investigation, software, and writing. A.S.: data curation, formal analysis, investigation, software, visualization, and writing. W.K.: formal analysis, investigation, writing. T.G.: formal analysis, supervision, investigation, visualization, and writing. All authors approved the final manuscript.

## Supplementary Material

giz094_GIGA-D-18-00504-Original-Submission

giz094_GIGA-D-18-00504_Revision-1

giz094_GIGA-D-18-00504_Revision-2

giz094_GIGA-D-18-00504_Revision-3

giz094_Response_to_Reviewer_Comments_Original_Submission

giz094_Response_to_Reviewer_Comments_Revision-1

giz094_Response_to_Reviewer_Comments_Revision_2

giz094_Reviewer_1_Report_Original_Submission Brent Pedersen -- 1/22/2019 Reviewed

giz094_Reviewer_1_Report_Revision_1 Brent Pedersen -- 6/4/2019 Reviewed

giz094_Reviewer_2_Report_Original_Submission Gianluigi Zanetti -- 2/10/2019 Reviewed

giz094_Reviewer_3_Report_Original_Submission Sara Goodwin -- 2/26/2019 Reviewed
