# Supplementary material for: SeQuiLa-cov: A fast and scalable library for depth of coverage calculations
Source: Gigascience. 2019 Aug 5;8(8):giz094. doi: 10.1093/gigascience/giz094 (PMC6680061; doi:10.1093/gigascience/giz094)
Supplement: giz094_GIGA-D-18-00504_Revision-1 [file giz094_giga-d-18-00504_revision-1.pdf]

GigaScience

SeQuiLa-cov: A fast and scalable library for depth of coverage calculations

--Manuscript Draft--

**Manuscript Number:**	GIGA-D-18-00504R1
**Full Title:**	SeQuiLa-cov: A fast and scalable library for depth of coverage calculations
**Article Type:**	Technical Note
**Funding Information:**	Ministerstwo Nauki i Szkolnictwa Wyższego (IP2015 019874) — Dr. Tomasz Gambin<br>Narodowe Centrum Nauki (2014/13/N/ST6/01843) — Mr. Marek Wiewiórka
**Abstract:**	Background: Depth of coverage calculation is an important and computationally intensive preprocessing step in a variety of next generation sequencing pipelines, including the analyses of RNA-seq data, detection of copy number variants, or quality control procedures.<br><br>Results: Building upon big data technologies, we have developed SeQuiLa-cov, an extension to the recently released SeQuiLa platform, which provides efficient depth of coverage calculations, reaching more than 100x speedup over the state-of-the-art tools. Performance and scalability of our solution allows for exome and genome-wide calculations running locally or on a cluster while hiding the complexity of the distributed computing with Structured Query Language Application Programming Interface.<br><br>Conclusions: SeQuiLa-cov provides significant performance gain in depth of coverage calculations streamlining the widely used bioinformatic processing pipelines.
**Corresponding Author:**	Tomasz Gambin<br>Warsaw University of Technology<br>Warszawa, POLAND
**Corresponding Author Secondary Information:**	
**Corresponding Author's Institution:**	Warsaw University of Technology
**Corresponding Author's Secondary Institution:**	
**First Author:**	Marek Wiewiórka
**First Author Secondary Information:**	
**Order of Authors:**	Marek Wiewiórka<br>Agnieszka Szmurło<br>Wiktor Kuśmirek<br>Tomasz Gambin
**Order of Authors Secondary Information:**	
**Response to Reviewers:**	May 24, 2019<br><br>The Editor of GigaScience<br><br>Dear Editor,<br><br>We are grateful to you and to Reviewers for the insightful comments, which have helped us to significantly improve our work. To address these comments, we have conducted additional experiments, updated our software (CRAM support, long reads support, Docker image enhancements, SciCrunch registration), made minor revisions to the main text and Project Documentation. Please, find below a detailed point-by-point response to all of the Reviewers' comments.

Most sincerely,
Tomasz Gambin, Ph. D.
ZSI-Bio group (http://biodatageeks.org)
Institute of Computer Science
Warsaw University of Technology
E-mail: tgambin@ii.pw.edu.pl

Reviewer 1

“1. In the intro the authors motivate the need for fast coverage. One example is that it is used by CNV callers. However, it's not clear to me that existing CNV callers could utilize the coverage information in SeQuiLa-cov as it's in their table format. Would it require export? Can tools other than SeQuiLa use the full coverage information available? How are the timings affected if export to a standard (BED) text format is required?”

We thank the Reviewer for this insightful comment. We agree that most of the state-of-the-art, non-distributed bioinformatics software (e.g. CNV callers, QC pipelines) are not capable of reading coverage data directly from tables using SQL. However, to make this information available to above mentioned tools the export of the coverage data to text format is not required. In fact, the tabular results of the coverage computations can be stored in various file formats, e.g. binary ones such as ORC, Parquet as well as text files such as CSV, TSV which can be further used by an external tool. We clarified output format options in the revised Functionality section.

We extended our experiments to include storing data in a single text file and assessed how this operation affects the overall performance. The results revealed that this action causes noticeable performance degradation predominantly due to additional stage of data collection to a single node. In the revised version of the Project Documentation we added a new section showing performance results (http://biodatageeks.org/sequila/benchmarking/benchmarking.html#performance-of-saving-coverage-results-as-a-single-bed-file) and instructions on how to store calculated coverage data as BED file (http://biodatageeks.org/sequila/fileformats/fileformats.html#bed).

Additionally in the revised manuscript we added new paragraph in the Conclusions section to underline that our primary focus is providing distributed pipelines for large-scale processing.
“Although the tool can be integrated with non-distributed software, our primary aim is to support large scale processing pipelines and the full advantage of SeQuiLa-cov’s scalability and performance will be available once it is deployed and executed in a distributed environment. We expect that there will be a growing number of scalable solutions (Big Data Genomics project [1] with tools DECA and Cannoli as well as GATK4 (https://software.broadinstitute.org/gatk/gatk4)) that can take advantage from reading input data directly from distributed storage systems.“

"2. Since scalability and speed are a key focus of the paper, the authors should show how this scales on CRAM data. Most centers and even small labs are moving to CRAM. In mosdepth, we show that we can get better speed with CRAM especially in --fast-mode."

As the Reviewer suggested in the revised version of the solution we added support for CRAM and performed additional experiments using this format. First we confirmed that scalability is preserved. However, we observed that timings for processing CRAM files are ~2.5 - 4 times higher than for BAM files (http://biodatageeks.org/sequila/benchmarking/benchmarking.html#cram-versus-bam-performance-comparison-for-wes-dataset-blocks).
Importantly, the processing times of the coverage calculation algorithm are equal for BAMs and CRAMs, however the reading stage is significantly slower in case of the latter one. To retrieve necessary information from the input files, SeQuiLa-cov utilizes external Hadoop-BAM library (https://github.com/HadoopGenomics/Hadoop-BAM). We also experimented with DisQ (https://github.com/disq-bio/disq), library for manipulating

bioinformatics formats in Apache Spark, also used by the newest GATK but conducted tests (data not shown) revealed that reading times did not differ noticeably. By no means this is important direction of our future work.

"3. From the benchmarking scripts here:
http://biodatageeks.org/sequila/benchmarking/benchmarking.html it's not clear
to me how the window mode was run with mosdepth. Much of the time in mosdepth in spent writing the per-base coverage which can be avoided if only the window information is needed by passing the `-n` flag."

We thank the Reviewer for pointing out this shortage in our Project Documentation. As suggested we included in the benchmarking scripts the command that was used to run mosdepth fixed-length window coverage. We were using the optimization flag ‘-n’ mentioned by the Reviewer. Sample command is as follows:

{time mos/mosdepth -n --by 500 win NA12878.proper.wes.bam;} 2>>
mos_wes_win_time_1.txt

All scripts used for benchmarking depth of coverage module are available at:
http://biodatageeks.org/sequila/benchmarking/benchmarking.html#depth-of-coverage.

"4. This is related to 1. But, given the relative youth of SeQuiLa, it would be good to have a clear statement about how/if SeQuiLa-cov results can be used outside of the SeQuiLa environment. If this is not feasible, that should be clear in the text. If use of these fast coverage results does not require buy-in to the full SeQuiLa ecosystem, that would be a boon for the software."

We thank the Reviewer for this valuable remark. We agree that it is important that functional modules of our solution are available independently, in the form of a command-line tool that supports existing data formats. In the revised version of our work we developed and published Docker image of SeQuiLa-cov. The image is publicly available at Docker hub (https://hub.docker.com/r/biodatageeks/bdg-sequila image: biodatageeks/bdg-sequila) and sample usage of depthOfCoverage tool (available inside the Docker container) is shown in the revised SeQuiLa Project Documentation
(http://biodatageeks.org/sequila/quickstart/quickstart.html#depthofcoverage-script). In the revised version of the manuscript in the Functionality section we also indicated the availability of the Docker image.

Regarding integration capabilities of SeQuiLa please refer to our response to comment 1.

Reviewer 2

"1. Since the goal of the paper is to present a new tool, I suggest to expand the functionality section to provide readers with some details on what they need to do to use the tool in practice."

We thank the Reviewer for this valuable comment. We added a new paragraph (Execution and integration options) to Functionality subsection in which we clarified the execution and integration possibilities of SeQuiLa-cov:

"SeQuiLa-cov can be used as an extension to Apache Spark in a form of external JAR dependency or can be executed from command-line as a Docker container. Both options can be run locally (on a single node) or on a Hadoop cluster using Yet Another Resource Negotiator (YARN). (See Project Documentation for sample commands). The tool accepts BAM/CRAM files as input and supports processing of short and long reads. The tabular output of the coverage computations can be stored in various file formats, e.g. binary (ORC, Parquet), as well as text (CSV, TSV). The tool can be integrated with state-of-the-art applications through text files, or can be used directly as an additional library in bioinformatics pipelines implemented in Scala, R, or Python. "

Additionally we significantly reorganized the Quickstart section of the Project Documentation to clarify and present examples of two main options of using SeQuiLa.

	We added sample commands for using SeQuiLa-cov Docker image and end-to-end instructions on how to use SeQuiLa-cov directly in spark-shell. (http://biodatageeks.org/sequila/quickstart/quickstart.html)<br><br>Finally we have prepared and included additional Use Case scenario in the Project Documentation showing coverage statistics "Generating coverage statistics for exons with the average depth-of-coverage > 20x (running on the Hadoop cluster)"<br><br>Reviewer 3<br><br>"1: The authors compare samtools per-base result to the the SeqQuiLa-cov blocks and fixed length results, showing a significant reduction in time. This is reasonable given that samtools has not event or window level output. However, SeQuiLa-cov has a base level output in addition to event and window. It would be useful of the authors could also include this comparison. "<br><br>We thank the Reviewer for this insightful comment. We performed suggested calculations i.e. SeQuiLa-cov on base level and added detailed results in the revised version of Project Documentation. The wall-time on a single core is comparable to the samtools' solution. We also re-confirmed the scalability of SeQuiLa-cov with the base level output and significant time reduction when executed in distributed environment (http://biodatageeks.org/sequila/benchmarking/benchmarking.html#base-level-coverage-performance-comparison-for-wes-dataset-with-samtools).<br><br>"2: Long-read sequencing is becoming a more important tool for WGS. Like with short read approaches, coverage is critical tool for a successful study. There has been very little work done on coverage calculator comparisons with long reads as a data input. It would be useful for the authors to discuss the performance of the tool on a long read data set."<br><br>As the Reviewer suggested, in the revised version of this work we performed additional experiment with aligned long WGS reads. This new dataset was added to Benchmarking section of Project Documentation http://biodatageeks.org/sequila/benchmarking/benchmarking.html#datasets-for-coverage-tests) We ensured that our solution handles coverage calculation from long reads (see: http://biodatageeks.org/sequila/architecture/architecture.html#distributed-depth-of-coverage)<br>We checked the quality as well as timings of the calculations and added these results to Project Documentation (http://biodatageeks.org/sequila/benchmarking/benchmarking.html#long-reads-support). We confirmed that the output was equal to the samtools' result. We also added a new section to Project Documentation showing the long reads processing (http://biodatageeks.org/sequila/usecases/usecases.html#nanopore-long-reads-from-wgs-analyses).<br><br>References:<br><br>Massie Mea. Adam: Genomics formats and processing patterns for cloud scale computing. University of California, Berkeley Technical Report, No UCB/EECS-2013 2013;207:2013.
**Additional Information:**	
**Question**	**Response**
Are you submitting this manuscript to a special series or article collection?	No
**Experimental design and statistics**	Yes

Powered by Editorial Manager® and ProduXion Manager® from Aries Systems Corporation

Full details of the experimental design and statistical methods used should be given in the Methods section, as detailed in our Minimum Standards Reporting Checklist. Information essential to interpreting the data presented should be made available in the figure legends.<br><br>Have you included all the information requested in your manuscript?	
**Resources**<br><br>A description of all resources used, including antibodies, cell lines, animals and software tools, with enough information to allow them to be uniquely identified, should be included in the Methods section. Authors are strongly encouraged to cite Research Resource Identifiers (RRIDs) for antibodies, model organisms and tools, where possible.<br><br>Have you included the information requested as detailed in our Minimum Standards Reporting Checklist?	Yes
**Availability of data and materials**<br><br>All datasets and code on which the conclusions of the paper rely must be either included in your submission or deposited in publicly available repositories (where available and ethically appropriate), referencing such data using a unique identifier in the references and in the "Availability of Data and Materials" section of your manuscript.<br><br>Have you have met the above requirement as detailed in our Minimum Standards Reporting Checklist?	Yes

Powered by Editorial Manager® and ProduXion Manager® from Aries Systems Corporation

Manuscript

Click here to view linked References

Click here to access/download;Manuscript;SeQuiLa_cov_tracked.pdf

OXFORD (GIGA)^n SCIENCE

GigaScience, 2018, 1–7

doi: xx.xxxx/xxxx
Manuscript in Preparation
Paper

PAPER

SeQuiLa-cov: A fast and scalable library for depth of coverage calculations

Marek Wiewiórka[1,*], Agnieszka Szmurło[1,*], Wiktor Kuśmirek[1] and Tomasz Gambin[1,†]

[1]Institute of Computer Science, Warsaw University of Technology, ul. Nowowiejska 15/19, 00-665 Warsaw, Poland,

[*]Contributed equally.
[†]Corresponding author.

Abstract

Background Depth of coverage calculation is an important and computationally intensive preprocessing step in a variety of next generation sequencing pipelines, including the analyses of RNA-seq data, detection of copy number variants, or quality control procedures. **Results** Building upon big data technologies, we have developed SeQuiLa-cov, an extension to the recently released SeQuiLa platform, which provides efficient depth of coverage calculations, reaching more than 100x speedup over the state-of-the-art tools. Performance and scalability of our solution allows for exome and genome-wide calculations running locally or on a cluster while hiding the complexity of the distributed computing with Structured Query Language Application Programming Interface. **Conclusions** SeQuiLa-cov provides significant performance gain in depth of coverage calculations streamlining the widely used bioinformatic processing pipelines.

Key words: NGS data analysis; depth of coverage; big data; distributed computing; SQL; CNV-calling; RNA-seq; quality control for sequencing data;

Findings

Introduction

Given a set of sequencing reads and a genomic contig, depth of coverage for a given position is defined as a total number of reads overlapping the locus.

The coverage calculation is a frequently performed but time-consuming step in the analysis of Next Generation Sequencing (NGS) data. In particular, Copy-Number Variant detection pipelines require obtaining sufficient read depth of the analyzed samples [1, 2, 3]. In other applications, the coverage is computed to assess the quality of the sequencing data (e.g. to calculate the percentage of genome with at least 30X read depth) or to identify genomic regions overlapped by insufficient number of reads for reliable variant calling [4]. Finally, depth of coverage is one of the most computationally intensive parts of differential expression analysis using RNA-seq data at single-base resolution [5, 6, 7].

A number of tools supporting this operation have been developed, with 22 of them specified in Omictools catalog [8]. Well known, state-of-the-art solutions include: samtools *depth* [9], bedtools *genomecov* [10], GATK *DepthOfCoverage* [11], sambamba [12], and mosdepth [13] (see comparison presented in Table 1).

Traditionally, these methods calculate the depth of coverage using a pileup-based approach (introduced in samtools [9] and used in GATK [11]), which is inefficient since it iterates through each nucleotide position at every read in a Binary Alignment Map (BAM) file. An optimized, event-based approach has been proposed in bedtools [10] and mosdepth [13]. These algorithms use only specific 'events', i.e. start and end of the alignment blocks within each read (Figure 1A) instead of analyzing every base of each read, which significantly reduces the overall computational complexity.

Samtools and bedtools depth of coverage modules do not

Compiled on: May 24, 2019.
Draft manuscript prepared by the author.

Key Points

- SeQuiLa-cov allows for high-coverage (~60x) genome-wide depth of coverage calculations in less than one minute.
- SeQuiLa-cov provides ANSI SQL compliant API for accessing and analyzing of aligned sequencing reads data.

Table 1. Comparison of leading coverage calculation software tools.

	Functionality				Implementation			
tool	approach	bases	blocks	windows	language	Intel GKL	parallelism type	interface
**samtools**	pileup	yes	no	no	C	no	none	cmd line
**bedtools**	events	yes	yes	no	C++	no	none	cmd line
**GATK**[1]	pileup	yes	no	no	Java	yes	distributed	cmd line
**sambamba**	pileup	no	yes	yes	D	no	multithreaded	cmd line
**mosdepth**	events	no	yes	yes	Nim	no	multithreaded [2]	cmd line
**SeQuiLa-cov**	events	yes	yes	yes	Scala	yes	distributed	Scala, SQL

[1]GATK *DepthOfCoverage* has not yet been ported to the latest version, i.e. GATK 4.x
[2]Only for BAM decompression

provide any support for multi-core environment. Mosdepth implements parallel BAM decompression, but its main algorithm remains sequential. Sambamba, on the other hand, promotes itself as a highly parallel tool, implementing depth of coverage calculations in a map-reduce fashion utilizing multiple threads on a single node. Regardless of parallelization degree, all of the above mentioned tools share a common bottleneck caused by using a single thread for returning results. Finally, GATK was the first genomic framework providing a support for distributed computations, however, the *DepthOfCoverage* method has not been ported yet to the current software release of the toolkit.

We present the first fully scalable, distributed, SQL-oriented solution designated for the depth of coverage calculations. SeQuiLa-cov, an extension to the recently released SeQuiLa [14] platform, runs a redesigned event-based algorithm for the distributed environment and provides convenient, SQL-compliant interface. ~~The tool can be easily integrated with other applications implemented in Scala, R, or Python.~~

Algorithm and implementation

Algorithm

Consider input data set, *read_set*, of aligned sequencing reads sorted by genomic position from a BAM file partitioned into the n data slices ($read\_set_1$, $read\_set_2$, ..., $read\_set_n$) (Figure 1B).

In the most general case, the algorithm can be used in a distributed environment where each cluster node computes the coverage for the subset of data slices using the event-based method. Specifically, for the i-th partition containing the set of reads ($read\_set_i$), the set of $events_{i,chr}$ vectors (where chr is an index of genomic contig represented in *read_set*) is allocated and updated, based on the items from $read\_set_i$. For all reads, the algorithm parses the CIGAR string and for each continuous alignment block characterized by *start* position and length *len* it increments by one the $events_{i,chr}(start)$ and decrements by one the value of $events_{i,chr}(start + len)$. To compute the partial coverage vector for partition i and contig chr, a vector value at the index j is calculated as follows:

$$partial\_coverage_{i,chr}(j) = \sum_{m=1}^{j} events_{i,chr}(m).$$

The result of this stage is a set of $partial\_coverage_{i,chr}$ vectors distributed among the computation nodes. To calculate the final coverage for the whole *read_set*, an additional step of correction for overlaps between the partitions is required. An overlap $overlap_{i,chr}$ of length l between vectors $partial\_coverage_{i,chr}$ and $partial\_coverage_{i+1,chr}$ may occur on the partition boundaries where l tailing genomic positions of $partial\_coverage_{i,chr}$ are the same as l heading genomic positions of $partial\_coverage_{i+1,chr}$ (see Figure 1C).

If an overlap is identified then the coverage values from the $partial\_coverage_{i,chr}$'s l-length tail are added into the $partial\_coverage_{i+1,chr}$'s head and subsequently the last l elements of $partial\_coverage_{i,chr}$ are removed. Once this correction step is completed, non-overlapping $coverage_{i,chr}$ vectors are collected and yield the final coverage values for the whole input *read_set*.

The main characteristic of the described algorithm is its ability to distribute data and calculations (such as BAM decompression and main coverage procedure) among the available computation nodes. Moreover, instead of simply performing full data reduction stage of the partial coverage vectors, our solution minimizes required data shuffling among cluster nodes by limiting it to the overlapping part of coverage vectors. Importantly, SeQuiLa-cov computation model supports fine-grained parallelism at user-defined partition size in contrary to the traditional, coarse-grained parallelization strategies that involve splitting input data at a contig level.

Implementation

We have implemented SeQuiLa-cov in Scala programming language using the Apache Spark framework. To efficiently access the data from a BAM file we have prepared a custom data source using Data Source Application Programming Interface (API) exposed by SparkSQL. Performance of the read operation benefits from the Intel Genomics Kernel Library (GKL) [15] used for decompressing the BAM files chunks and from predicate push-down mechanism that filters out data at the earliest stage.

The implementation of the core coverage calculation algorithm aimed at minimizing, whenever possible memory footprint by using parsimonious data types, e.g. *Short* type instead of *Integer*, and efficient memory allocation strategy for large data structures, e.g. favoring static *Arrays* over dynamic size *ArrayBuffers*. Additionally, to reduce the overhead of data shuffling between the worker nodes in the correction for the overlaps stage we used Spark's shared variables [16] *accumulators* and *broadcast variables* (Figure 1C). Accumulator is used to gather information about the worker nodes' coverage vector ranges and coverage vector tail values, that are subsequently read and processed by the driver. This information is then used to construct a broadcast variable distributed to the worker nodes in order to perform adequate trimming and summing operations on partial coverage vectors.

Table 2. Benchmarking leading solutions against SeQuiLa-cov on WES/WGS data in performing blocks and windows calculations

data	operation type	cores	samtools	bedtools	sambamba	mosdepth	SeQuiLa-cov
WGS	blocks	1	2h 14m 58s [1]	10h 41m 27s	2h 44m 0s	**1h 46m 27s**	1h 47m 5s
		5			2h 47m 53s	36m 13s	**26m 59s**
		10			2h 50m 47s	34m 34s	**13m 54s**
	fixed-length windows	1			1h 46m 50s	**1h 22m 49s**	1h 24m 8s
		5			1h 41m 23s	20m 3s	**18m 43s**
		10			1h 50m 35s	17m 49s	**9m 14s**
WES	blocks	1	12m 26s [1]	23m 25s	25m 42s	**6m 43s**	6m 54s
		5			25m 46s	2m 25s	**1m 47s**
		10			25m 49s	2m 20s	**1m 4s**
	fixed-length windows	1			14m 36s	**6m 11s**	6m 29s
		5			14m 54s	2m 8s	**1m 42s**
		10			14m 40s	2m 14s	**1m 1s**

Both samtools and bedtools calculate coverage using only a single thread, however, their results differ significantly, with samtools being around twice as fast. Sambamba positions itself as a multithreaded solution although our tests revealed that its execution time is nearly constant, regardless of the number of CPU cores used, and even twice as slow as samtools. Mosdepth achieved speedup against samtools in blocks coverage and against sambamba in windows coverage calculations, however, its scalability reaches limit at 5 CPU cores. Finally, SeQuiLa-cov, achieves nearly identical performance as mosdepth for the single core but the execution time decreases substantially for greater number of available computing resources which makes this solution the fastest when run on multiple cores and nodes.
[1]per-base results are treated as blocks output. Samtools lacks the functionality of blocks coverage calculations, however, we included this tool in our benchmark for completeness, treating its per-base results as blocks outcome assuming that both result types require nearly the same resources.

Functionality

Supported coverage result types

SeQuiLa-cov features three distinct result types: *per-base*, *blocks*, and *fixed-length windows* coverage (Figure 1A). For *per-base*, the depth of coverage is calculated and returned for each genomic position making it the most verbose output option. The method producing block level coverage (*blocks*) involves merging adjacent genomic positions with equal coverage values into genomic intervals. As a consequence, fewer records than in case of *per-base* output type are generated with no information loss. The *fixed-length windows* the algorithm generates set of fixed length, tiling, non-overlapping genomic intervals and returns arithmetic mean of coverage values over positions within each window.

ANSI SQL compliance

SeQuiLa-cov solution promotes SQL as a data query and manipulation language in genomic analysis. Data flows are performed in SQL-like manner through the custom data source supporting convenient Create Table as Select and Insert as Select methods. SeQuiLa-cov provides a table abstraction over existing alignment ~~BAM/CRAM~~ files, with no need of data conversion, which can be further ~~conveniently~~ queried and manipulated in a declarative way. The coverage calculation function *bdg_coverage*, as described in Algorithm sub-section, has been implemented as *table-valued function*(Figure 1D).

Execution and integration options

SeQuiLa-cov can be used as an extension to Apache Spark in a form of external JAR dependency or can be executed from command-line as a Docker container. Both options can be run locally (on a single node) or on a Hadoop cluster using Yet Another Resource Negotiator (YARN). (See Project Documentation for sample commands). The tool accepts BAM/CRAM files as input and supports processing of short and long reads. The tabular output of the coverage computations can be stored in various file formats, e.g. binary (ORC, Parquet), as well as text (CSV, TSV). The tool can be integrated with state-of-the-art applications through text files, or can be used directly as an additional library in bioinformatics pipelines implemented in Scala, R, or Python.

Benchmarking

We have benchmarked SeQuiLa-cov solution with leading software for depth of coverage calculations, specifically samtools *depth*, bedtools *genomeCov*, sambamba *depth* and mosdepth (results of *DepthOfCOverage* from outdated GATK version are available in supplementary data). The tests were performed on the aligned WES and WGS reads from the NA12878 sample (see Methods for details) and aimed at calculating blocks and window coverage. To compare the performance and scalability of each solution, we have executed calculations for 1, 5, and 10 cores on a single computation node (see Table 2).

Samtools *depth* and bedtools *genomeCov* are both natively non-scalable and were run on a single thread only. Exome-wide calculations exceeded 10 minutes and genome-wide analyses took over two hours in case of samtools, while bedtools' performance was significantly worse, i.e ~1.9x for WES and ~4.75x for WGS. Sambamba *depth* declares to take advantage of fully parallelized data processing with the use of multithreading. However, our results revealed that even when additional threads were used, the total execution time of coverage calculations remained nearly constant and greater than samtools's result. Mosdepth shows significant speedup (~1.3x) against samtools when using single thread. This performance gain increases to ~3.7x when using 5 decompression threads, however, it does not benefit from adding additional CPU power. In case of fixed-length window coverage mosdepth achieves over ~1.3 speedup against sambamba.

SeQuiLa-cov achieves performance similar to mosdepth when run using a single core. However, SeQuiLa-cov is ~1.3x and ~2.5x as fast as mosdepth when using 5 and 10 CPU cores, respectively, demonstrates its better scalability. The similar performance characteristic is observed for both blocks and fixed-length windows methods.

To fully assess the scalability profile of our solution, we have performed additional tests in a cluster environment (see Methods for details). Our results show that when utilizing additional resources (i.e. more than 10 CPU cores), SeQuiLa-cov is able to reduce the total computation time to 15 seconds for WES and less than one minute for WGS data (Figure 2). Scalability limit is achieved for 200 and ~500 CPU cores in case of WES and WGS data, respectively.

To evaluate the impact of Intel GKL library on deflate operation (BAM bzgf block decompression), we have performed blocks coverage calculations on WES data on 50 CPU cores. The

results showed on average ~1.18x speedup when running with Intel GKL deflate implementation.

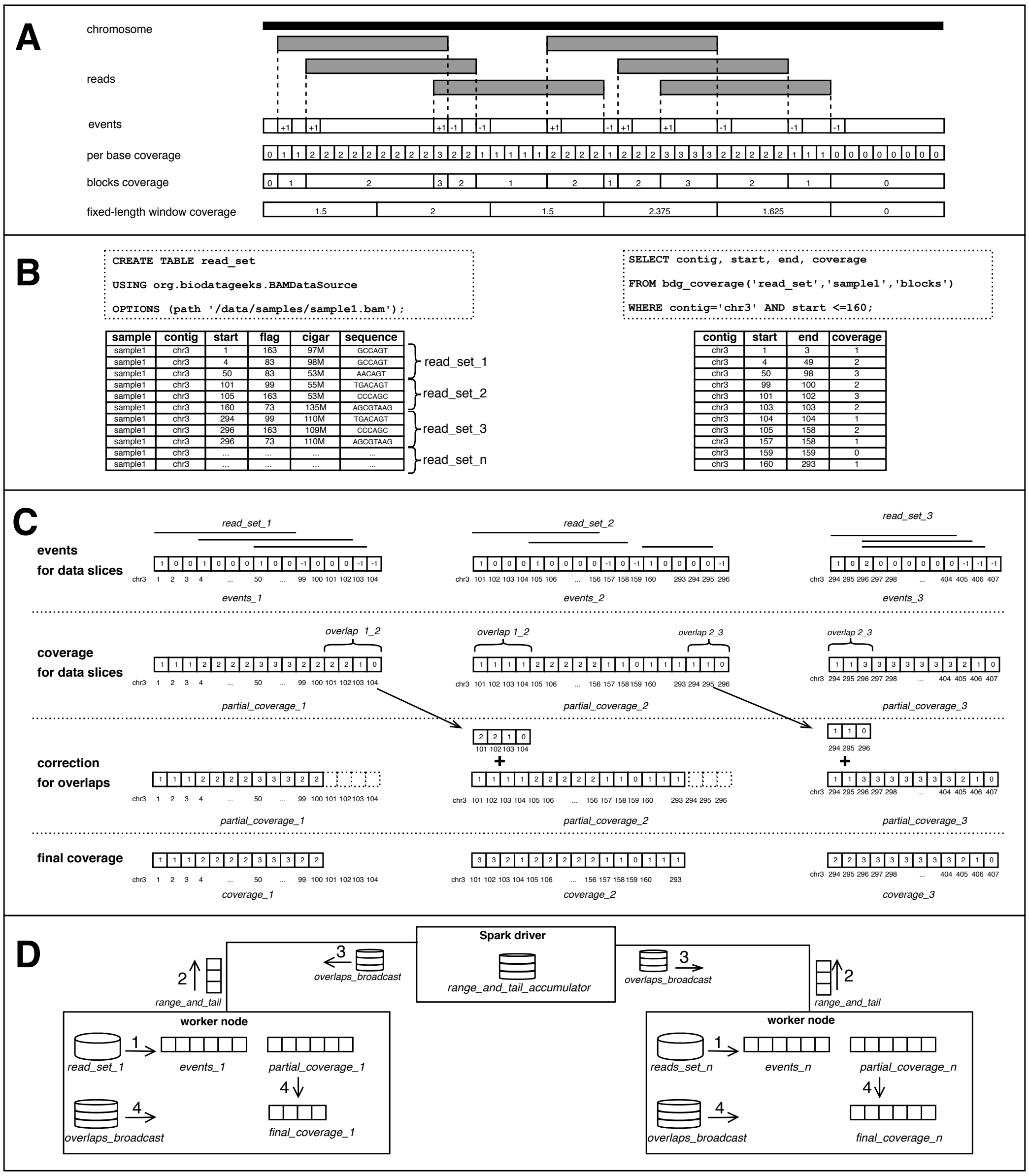

Figure 1. SeQuiLa-cov: functionality, algorithm and implementation

Panel A shows the general concept of events-based algorithm for depth of coverage calculation. Given a genomic chromosome and a set of aligned sequencing reads, the algorithm allocates *events* vector. Subsequently, it iterates the list of reads and increments/decrements by one the values of the *events* vector at the indexes corresponding to start/end positions of each read. The depth of coverage for a genomic locus is calculated using the cumulative sum of all elements in the *events* vector preceding specified position. The algorithm may produce three typically used coverage types: (i) *per-base* coverage, which includes the coverage value for each genomic position separately, (ii) *blocks* which lists adjacent positions with equal coverage values are merged into single interval, and (iii) *fixed-length windows* coverage that generates set of equal-size, non-overlapping and tiling genomic ranges and outputs arithmetic mean of base coverage values for each region.

Panel B presents the provided SQL API to interact with NGS data. The first statement creates a relational table *read_set* over compressed BAM files using the provided custom Data Source, whereas the second statement demonstrates the use of *bdg_coverage* function to calculate depth of coverage for a specified sample. The presented call for coverage method takes sample identifier (sample1) and result type (blocks) as input parameters. *bdg_coverage* is implemented as a table-valued function. Therefore, it outputs a table as a result allowing for customizing a query using Data Manipulation Language e.g. in the SELECT or WHERE clause. For the purpose of this example, we assume that BAM file for sample1 contains only reads from chr3.

Panel C shows the concept of distributed version of events-based algorithm. Assuming that we run our calculations in a distributed environment, the computation nodes do not work on the whole input data set (table *read_set*) but on *n* smaller data partitions ($slice_1$, $slice_2$, .. ,$slice_n$), each containing subset of input aligned reads. First the algorithm calculates partial *events* vector for available data slices and subsequently produces corresponding partial *partial_coverage* vector. Due to the possibility of overlapping of ranges between two consecutive data slices, additional correction step needs to be performed. When an overlap is identified, the corresponding coverage values from the preceding vector's tail are cut and added to the head values of the subsequent vector. On the figure two overlaps were shown, one of them situated between $partial\_coverage_1$ and $partial\_coverage_2$ ($overlap_{12}$ of length 4) encompassing positions chr3:101-104. The coverage values from $partial\_coverage_1$ for $overlap_{12}$ are removed from $partial\_coverage_1$ and added to the head of $partial\_coverage_2$. As a result, a set of non-overlapping coverage vectors are calculated, which is further integrated into the depth of coverage for the whole input data set.

Panel D presents the implementation details of SeQuiLa-cov. We have used the Apache Spark environment, where a single driver node runs the high-level driver program, which schedules tasks for multiple worker nodes. On each worker node, a set of data partitions are accessed and manipulated in order to generate *events* and *partial_coverage* vectors. To gather data about *partial_coverage* vectors' ranges along with tailing coverage values, and to distribute data needed for rearranging coverage vector values and ranges, we have used Spark's shared variables *accumulator* and *broadcast*, respectively.

Finally, our comprehensive functional unit testing showed that results calculated by SeQuiLa-cov and samtools *depth* are identical.

Conclusions

The application of the recent advancements in big data technologies and distributed computing can contribute to both speeding up genomic data processing and management. Analysis of large genomic data sets require efficient, accurate, and scalable algorithms to perform calculations utilizing the computing power of multiple cluster nodes. In this work, we show that with sufficiently large cluster genome-wide coverage calculations may last less than a minute and at the same time being over 100x faster than the best single-threaded solution.

Although the tool can be integrated with non-distributed software, our primary aim is to support large scale processing pipelines and the full advantage of SeQuiLa-cov's scalability and performance will be available once it is deployed and executed in a distributed environment. We expect that there will be a growing number of scalable solutions (Big Data Genomics project [17] with tools DECA and Cannoli as well as GATK4 (https://software.broadinstitute.org/gatk/gatk4)) that can take advantage of reading input data directly from distributed storage systems.

SeQuiLa-cov is one of the building blocks of SeQuiLa [14] ecosystem, which initiated the move towards efficient, distributed processing of genomic data and providing SQL-oriented API for convenient and elastic querying. We foresee that following this direction will enable the evolution of genomic data analysis from the file-oriented to table-oriented processing.

Methods

Test data

We have tested our solution using reads from NA12878 sample which were aligned to hg18 genome. WES data containing over 161 million of reads weights 17 GB and WGS data include over 2,6 billion of reads taking 272 GB of disk space. Both BAM files were compressed at the default BAM's compression level (5).

Testing environment

To perform comprehensive performance evaluation, we have setup a test cluster consisting of 28 Hadoop nodes (1 edge node, 3 master nodes and 24 data nodes) with Hortonworks Data Platform 3.0.1 installed. Each data node has 28 cores (56 with hyper-threading) and 512 GB of RAM, YARN ~~Yet Another Resource Negotiator (YARN)~~ resource pool has been configured with 2640 virtual cores and 9671 GB RAM.

Investigated solutions

In our benchmark we have used the most recent versions of the investigated tools i.e. samtools version 1.9, bedtools 2.27.0, sambamba 0.6.8, mosdepth version 0.2.3 and SeQuiLa-cov version 0.5.1.

Availability of source code and requirements

- Project name: SeQuiLa-cov
- Project home page: http://biodatageeks.org/sequila/
- Source code repository: https://github.com/ZSI-Bio/bdg-sequila
- Operating system: Platform independent
- Programming language: Scala
- Other requirements: Docker
- License: Apache License 2.0

Availability of supporting data and materials

The Docker image is available at https://hub.docker.com/r/biodatageeks/. Supplementary information on benchmarking procedure as well as test data ~~is~~are publicly accessible at project documentation site http://biodatageeks.org/sequila/benchmarking/benchmarking.html#depth-of-coverage. The resource is available at SciCrunch: SeQuiLa, RRID:SCR_017220 .

Declarations

List of abbreviations

API – Application Programming Interface
BAM – Binary Alignment Map
GKL – Genomics Kernel Library
NGS – Next Generation Sequencing
SQL – Structured Query Language
YARN – Yet Another Resource Negotiator
WES – Whole Exome Sequencing
WGS – Whole Genome Sequencing

Consent for publication

Not applicable

Competing interests

None of the authors have any competing interests.

Funding

This work has been supported by the Polish budget funds for science in years 2016–2019 (Iuventus Plus grant IP2015 019874), as well as Polish National Science Center grant Preludium 2014/13/N/ST6/01843.

Author's Contributions

MW – conceptualization, formal analysis, investigation, software and writing. AS – data curration, formal analysis, investigation, software, visualization and writing. WK – formal analysis, investigation, writing. TG – formal analysis, supervision, investigation, visualization and writing. All authors approved the final manuscript.

References

1. Fromer M, Purcell SM. Using XHMM Software to Detect Copy Number Variation in Whole-Exome Sequencing Data. Current protocols in human genetics 2014 4;81:1–21. http://www.ncbi.nlm.nih.gov/pubmed/24763994http://www.pubmedcentral.nih.gov/articlerender.fcgi?artid=PMC4065038.

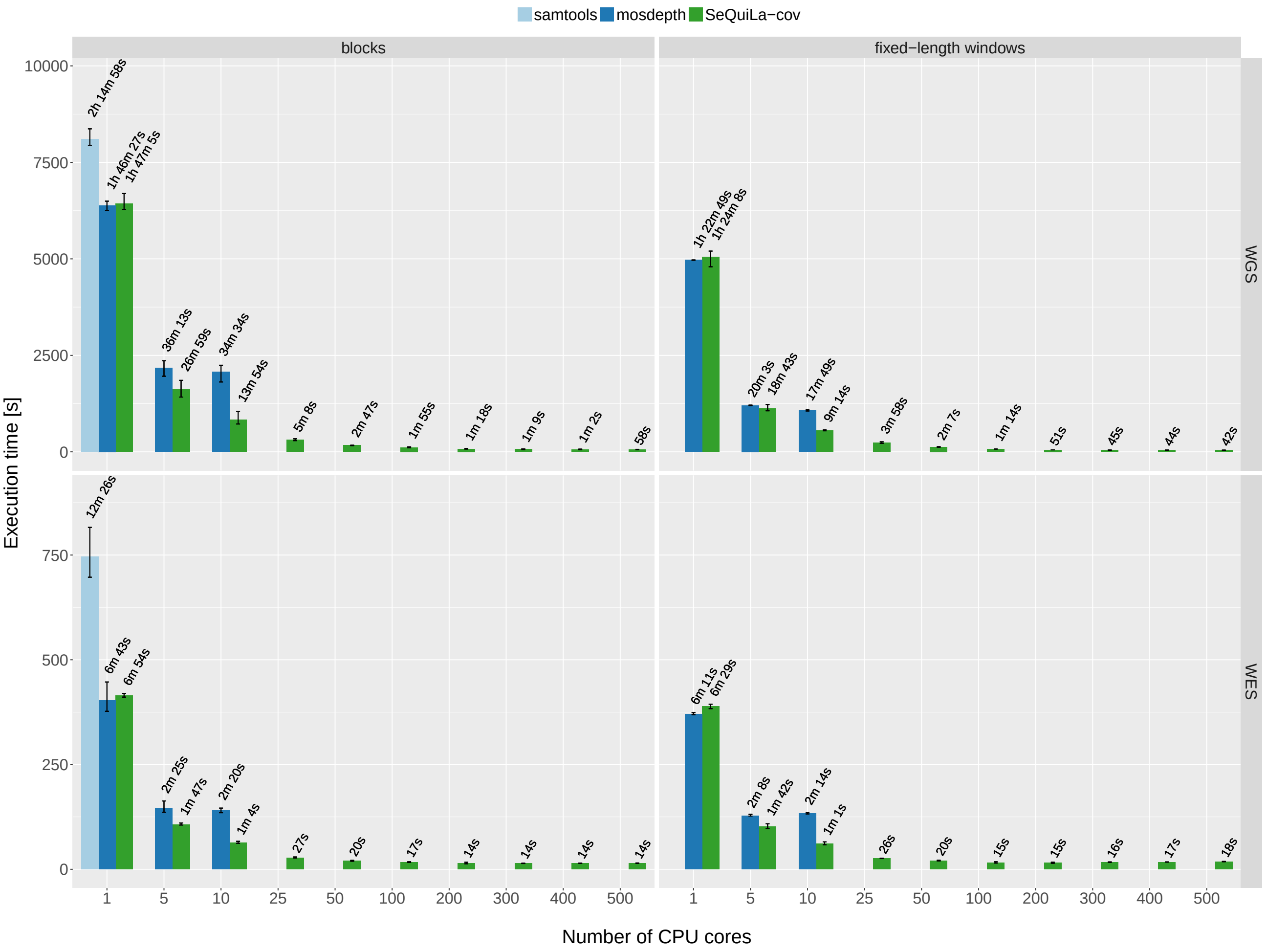

Figure 2. Performance and scalability comparison of samtools, mosdepth and SeQuiLa-cov

Each experiment setting was repeated several times and the height of each bar along with the corresponding error bars indicate the average, as well as the minimum and maximum execution time, respectively. The best pileup-based solution is definitely slower (two times for WGS calculations) than both event-based solutions what clearly shows the superiority of the latter one. Mosdepth execution time scales up to 5 cores, afterwards it shows no furthe gain in performance. SeQuiLa-cov has nearly the same execution time results as mosdepth for both blocks and windows calculations for a single core, but scales out desirably utilizing all 500 CPU cores on cluster nodes and at the same time performing WGS calculations in less than 1 minute.

2. Jiang Y, Oldridge DA, Diskin SJ, Zhang NR. CODEX: a normalization and copy number variation detection method for whole exome sequencing. Nucleic acids research 2015 3;43(6):e39. http://www.ncbi.nlm.nih.gov/pubmed/25618849http://www.pubmedcentral.nih.gov/articlerender.fcgi?artid=PMC4381046.
3. Gambin T, Akdemir ZC, Yuan B, Gu S, Chiang T, Carvalho CMB, et al. Homozygous and hemizygous CNV detection from exome sequencing data in a Mendelian disease cohort. Nucleic acids research 2017;45(4):1633–1648. http://www.ncbi.nlm.nih.gov/pubmed/27980096http://www.pubmedcentral.nih.gov/articlerender.fcgi?artid=PMC5389578.
4. Okonechnikov K, Conesa A, García-Alcalde F. Qualimap 2: advanced multi-sample quality control for high-throughput sequencing data. Bioinformatics 2015 10;32(2):btv566. https://academic.oup.com/bioinformatics/article-lookup/doi/10.1093/bioinformatics/btv566.
5. Frazee AC, Sabunciyan S, Hansen KD, Irizarry RA, Leek JT. Differential expression analysis of RNA-seq data at single-base resolution. Biostatistics 2014 7;15(3):413–426. https://academic.oup.com/biostatistics/article-lookup/doi/10.1093/biostatistics/kxt053.
6. Nellore A, Collado-Torres L, Jaffe AE, Alquicira-Hernández J, Wilks C, Pritt J, et al. Rail-RNA: scalable analysis of RNA-seq splicing and coverage. Bioinformatics 2016 9;33(24):btw575. https://academic.oup.com/bioinformatics/article-lookup/doi/10.1093/bioinformatics/btw575.
7. Collado-Torres L, Nellore A, Frazee AC, Wilks C, Love MI, Langmead B, et al. Flexible expressed region analysis for RNA-seq with derfinder. Nucleic Acids Research 2017 1;45(2):e9–e9. https://academic.oup.com/nar/article-lookup/doi/10.1093/nar/gkw852.
8. Coverage/Depth analysis bioinformatics tools | Next-generation sequencing analysis - OMICtools;. https://omictools.com/depth-of-coverage-category.
9. Li Hea. The Sequence Alignment/Map format and SAMtools. Bioinformatics 2009 8;25(16):2078–2079.
10. Quinlan AR, Hall IM. BEDTools: a flexible suite of utilities for comparing genomic features. Bioinformatics (Oxford, England) 2010 3;26(6):841–2. http://www.ncbi.nlm.nih.gov/pubmed/20110278http://www.pubmedcentral.nih.gov/articlerender.fcgi?artid=PMC2832824.
11. McKenna A, Hanna M, Banks E, Sivachenko A, Cibulskis K, Kernytsky A, et al. The Genome Analysis Toolkit: a MapReduce framework for analyzing next-generation DNA sequencing data. Genome research 2010 9;20(9):1297–303. http://www.ncbi.nlm.nih.gov/pubmed/20644199http://www.pubmedcentral.nih.gov/articlerender.fcgi?artid=PMC2928508.
12. Tarasov A, Vilella AJ, Cuppen E, Nijman IJ, Prins P. Sambamba: fast processing of NGS alignment formats. Bioinformatics (Oxford, England) 2015 6;31(12):2032–4. http://www.ncbi.nlm.nih.gov/pubmed/25697820http://www.pubmedcentral.nih.gov/articlerender.fcgi?artid=PMC4765878.
13. Pedersen BS, Quinlan AR. Mosdepth: quick coverage calculation for genomes and exomes. Bioinformatics 2018 3;34(5):867–868. http://www.ncbi.nlm.nih.gov/pubmed/29096012http://www.pubmedcentral.nih.gov/articlerender.fcgi?artid=PMC6030888https://academic.oup.com/bioinformatics/article/34/5/867/4583630.
14. Wiewiórka M, Leśniewska A, Szmurło A, Stępień K, Borowiak M, Okoniewski M, et al. SeQuiLa: An elastic, fast and scalable SQL-oriented solution for processing and querying genomic intervals. Bioinformatics 2018 11;https://academic.oup.com/bioinformatics/advance-article/doi/10.1093/bioinformatics/bty940/5182295.
15. James Guilford A, Powley G, Tucker G, Vaidya P, Bergelson L, Lichtenstein L, et al. Accelerating the Compression and Decompression of Genomics Data using GKL Provided by Intel; 2017, https://www.intel.com/content/dam/www/public/us/en/documents/white-papers/accelerating-genomics-data-gkl-white-paper.pdf.
16. Zaharia M, Chowdhury M, J Franklin M, Shenker S, Stoica I. Spark: Cluster Computing with Working Sets. Proceedings of the 2nd USENIX conference on Hot topics in cloud computing 2010 12;10:10. https://www.usenix.org/legacy/event/hotcloud10/tech/full_papers/Zaharia.pdf.
17. Massie Mea. Adam: Genomics formats and processing patterns for cloud scale computing. University of California, Berkeley Technical Report, No UCB/EECS-2013 2013;207:2013.
