# Supplementary material for: SeQuiLa-cov: A fast and scalable library for depth of coverage calculations
Source: Gigascience. 2019 Aug 5;8(8):giz094. doi: 10.1093/gigascience/giz094 (PMC6680061; doi:10.1093/gigascience/giz094)
Supplement: giz094_Response_to_Reviewer_Comments_Original_Submission [file giz094_response_to_reviewer_comments_original_submission.pdf]

Author's Response To Reviewer Comments

Close

May 24, 2019

The Editor of GigaScience

Dear Editor,

We are grateful to you and to Reviewers for the insightful comments, which have helped us to significantly improve our work. To address these comments, we have conducted additional experiments, updated our software (CRAM support, long reads support, Docker image enhancements, SciCrunch registration), made minor revisions to the main text and Project Documentation. Please, find below a detailed point-by-point response to all of the Reviewers' comments.

Most sincerely,
Tomasz Gambin, Ph. D.
ZSI-Bio group (http://biodatageeks.org)
Institute of Computer Science
Warsaw University of Technology
E-mail: tgambin@ii.pw.edu.pl

Reviewer 1

"1. In the intro the authors motivate the need for fast coverage. One example is that it is used by CNV callers. However, it's not clear to me that existing CNV callers could utilize the coverage information in SeQuiLa-cov as it's in their table format. Would it require export? Can tools other than SeQuiLa use the full coverage information available? How are the timings affected if export to a standard (BED) text format is required?"

We thank the Reviewer for this insightful comment. We agree that most of the state-of-the-art, non-distributed bioinformatics software (e.g. CNV callers, QC pipelines) are not capable of reading coverage data directly from tables using SQL. However, to make this information available to above mentioned tools the export of the coverage data to text format is not required. In fact, the tabular results of the coverage computations can be stored in various file formats, e.g. binary ones such as ORC, Parquet as well as text files such as CSV, TSV which can be further used by an external tool. We clarified output format options in the revised Functionality section.

We extended our experiments to include storing data in a single text file and assessed how this operation affects the overall performance. The results revealed that this action causes noticeable performance degradation predominantly due to additional stage of data collection to a single node. In the revised version of the Project Documentation we added a new section showing performance results (http://biodatageeks.org/sequila/benchmarking/benchmarking.html#performance-of-saving-coverage-results-as-a-single-bed-file) and instructions on how to store calculated coverage data as BED file (http://biodatageeks.org/sequila/fileformats/fileformats.html#bed).

Additionally in the revised manuscript we added new paragraph in the Conclusions section to underline that our primary focus is providing distributed pipelines for large-scale processing.
"Although the tool can be integrated with non-distributed software, our primary aim is to support large scale processing pipelines and the full advantage of SeQuiLa-cov's scalability and performance will be available once it is deployed and executed in a distributed environment. We expect that there will be a growing number of scalable solutions (Big Data Genomics project [1] with tools DECA and Cannoli as well as GATK4 (https://software.broadinstitute.org/gatk/gatk4)) that can take advantage from reading input data directly from distributed storage systems."

"2. Since scalability and speed are a key focus of the paper, the authors should show how this scales on CRAM data. Most centers and even small labs are moving to CRAM. In mosdepth, we show that we can get better speed with CRAM especially in --fast-mode."

As the Reviewer suggested in the revised version of the solution we added support for CRAM and performed additional experiments using this format. First we confirmed that scalability is preserved. However, we observed that timings for processing CRAM files are ~2.5 - 4 times higher than for BAM files (http://biodatageeks.org/sequila/benchmarking/benchmarking.html#cram-versus-bam-performance-comparison-for-wes-dataset-blocks).
Importantly, the processing times of the coverage calculation algorithm are equal for BAMs and CRAMs, however the reading stage is significantly slower in case of the latter one. To retrieve necessary information from the input files, SeQuiLa-cov utilizes external Hadoop-BAM library (https://github.com/HadoopGenomics/Hadoop-BAM). We also experimented with DisQ (https://github.com/disq-bio/disq), library for manipulating bioinformatics formats in Apache Spark, also used by the newest GATK but conducted tests (data not shown) revealed that reading times did not differ noticeably. By no means this is important direction of our future work.

"3. From the benchmarking scripts here:
http://biodatageeks.org/sequila/benchmarking/benchmarking.html it's not clear
to me how the window mode was run with mosdepth. Much of the time in mosdepth in spent writing the per-base coverage which can be avoided if only the window information is needed by passing the `-n` flag."

We thank the Reviewer for pointing out this shortage in our Project Documentation. As suggested we included in the benchmarking scripts the command that was used to run mosdepth fixed-length window coverage. We were using the optimization flag ‘-n’ mentioned by the Reviewer. Sample command is as follows:

{time mos/mosdepth -n --by 500 win NA12878.proper.wes.bam;} 2>> mos_wes_win_time_1.txt

All scripts used for benchmarking depth of coverage module are available at:
http://biodatageeks.org/sequila/benchmarking/benchmarking.html#depth-of-coverage.

"4. This is related to 1. But, given the relative youth of SeQuiLa, it would be good to have a clear statement about how/if SeQuiLa-cov results can be used outside of the SeQuiLa environment. If this is not feasible, that should be clear in the text. If use of these fast coverage results does not require buy-in to the full SeQuiLa ecosystem, that would be a boon for the software."

We thank the Reviewer for this valuable remark. We agree that it is important that functional modules of our solution are available independently, in the form of a command-line tool that supports existing data formats. In the revised version of our work we developed and published Docker image of SeQuiLa-cov. The image is publicly available at Docker hub (https://hub.docker.com/r/biodatageeks/bdg-sequila image: biodatageeks/bdg-sequila) and sample usage of depthOfCoverage tool (available inside the Docker container) is shown in the revised SeQuiLa Project Documentation (http://biodatageeks.org/sequila/quickstart/quickstart.html#depthofcoverage-script). In the revised version of the manuscript in the Functionality section we also indicated the availability of the Docker image.

Regarding integration capabilities of SeQuiLa please refer to our response to comment 1.

Reviewer 2

"1. Since the goal of the paper is to present a new tool, I suggest to expand the functionality section to provide readers with some details on what they need to do to use the tool in practice."

We thank the Reviewer for this valuable comment. We added a new paragraph (Execution and integration options) to Functionality subsection in which we clarified the execution and integration possibilities of SeQuiLa-cov:

"SeQuiLa-cov can be used as an extension to Apache Spark in a form of external JAR dependency or can be executed from command-line as a Docker container. Both options can be run locally (on a single node) or on a Hadoop cluster using Yet Another Resource Negotiator (YARN). (See Project

Documentation for sample commands). The tool accepts BAM/CRAM files as input and supports processing of short and long reads. The tabular output of the coverage computations can be stored in various file formats, e.g. binary (ORC, Parquet), as well as text (CSV, TSV). The tool can be integrated with state-of-the-art applications through text files, or can be used directly as an additional library in bioinformatics pipelines implemented in Scala, R, or Python. "

Additionally we significantly reorganized the Quickstart section of the Project Documentation to clarify and present examples of two main options of using SeQuiLa.
We added sample commands for using SeQuiLa-cov Docker image and end-to-end instructions on how to use SeQuiLa-cov directly in spark-shell. (http://biodatageeks.org/sequila/quickstart/quickstart.html)

Finally we have prepared and included additional Use Case scenario in the Project Documentation showing coverage statistics "Generating coverage statistics for exons with the average depth-of-coverage > 20x (running on the Hadoop cluster)"

Reviewer 3

"1: The authors compare samtools per-base result to the the SeqQuiLa-cov blocks and fixed length results, showing a significant reduction in time. This is reasonable given that samtools has not event or window level output. However, SeQuiLa-cov has a base level output in addition to event and window. It would be useful of the authors could also include this comparison. "

We thank the Reviewer for this insightful comment. We performed suggested calculations i.e. SeQuiLa-cov on base level and added detailed results in the revised version of Project Documentation. The wall-time on a single core is comparable to the samtools' solution. We also re-confirmed the scalability of SeQuiLa-cov with the base level output and significant time reduction when executed in distributed environment (http://biodatageeks.org/sequila/benchmarking/benchmarking.html#base-level-coverage-performance-comparison-for-wes-dataset-with-samtools).

"2: Long-read sequencing is becoming a more important tool for WGS. Like with short read approaches, coverage is critical tool for a successful study. There has been very little work done on coverage calculator comparisons with long reads as a data input. It would be useful for the authors to discuss the performance of the tool on a long read data set."

As the Reviewer suggested, in the revised version of this work we performed additional experiment with aligned long WGS reads. This new dataset was added to Benchmarking section of Project Documentation http://biodatageeks.org/sequila/benchmarking/benchmarking.html#datasets-for-coverage-tests) We ensured that our solution handles coverage calculation from long reads (see: http://biodatageeks.org/sequila/architecture/architecture.html#distributed-depth-of-coverage)
We checked the quality as well as timings of the calculations and added these results to Project Documentation (http://biodatageeks.org/sequila/benchmarking/benchmarking.html#long-reads-support). We confirmed that the output was equal to the samtools' result. We also added a new section to Project Documentation showing the long reads processing (http://biodatageeks.org/sequila/usecases/usecases.html#nanopore-long-reads-from-wgs-analyses).

References:

Massie Mea. Adam: Genomics formats and processing patterns for cloud scale computing. University of California, Berkeley Technical Report, No UCB/EECS-2013 2013;207:2013.

Close
