# Supplementary material for: SeQuiLa-cov: A fast and scalable library for depth of coverage calculations
Source: Gigascience. 2019 Aug 5;8(8):giz094. doi: 10.1093/gigascience/giz094 (PMC6680061; doi:10.1093/gigascience/giz094)
Supplement: giz094_Response_to_Reviewer_Comments_Revision-1 [file giz094_response_to_reviewer_comments_revision-1.pdf]

Author's Response To Reviewer Comments

Close

June 10, 2019

The Editor of GigaScience

Dear Editor,

Thank you for accepting our manuscript for publication in GigaScience. Please, find below a detailed point-by-point response to all of your comments.

Most sincerely,
Tomasz Gambin, Ph. D.
ZSI-Bio group (http://biodatageeks.org)
Institute of Computer Science
Warsaw University of Technology
E-mail: tgambin@ii.pw.edu.pl

1) Our data curators usually take an archival snapshot of the source code and test data, which will be posted via our repository GigaDB. Our curators will contact you shortly to prepare this dataset.
Once this is ready, you will get a DOI for this GigaDB dataset. Please include a citation to your GigaDB dataset (including the DOI link) to your reference list, and cite this in the data availability section in the manuscript, where appropriate.
Please follow this example format for the reference:
[xx] Author1 N, Author2 N, AuthorX N. Supporting data [and code] for "Title of your manuscript". GigaScience Database. 2019. http://dx.doi.orgxxxxxxxxxxxx

We are looking forward to contacting us by data curators.

2) I note that all authors are also listed as "corresponding author" on the title page. Gigascience allows a maximum of two corresponding authors (see https://academic.oup.com/gigascience/pages/authorship_guidelines) - please discuss who (one or two authors) should take on this role.

We double checked this and there is only one corresponding author (Tomasz Gambin) indicated on the title page. Please, let us know if you need us to change the way how the corresponding author is marked.

3) To avoid confusion during the production process, please remove any highlighting / tracking of changes that was made for the purpose of peer review.

We removed all highlighting as requested.

4) Thank you for registering your tool at Scicrunch. The RRID should be mentioned as the last bullet point in the "software availability" section (below "licence"), in the following format:
RRID: SCR_NNNNN
(see https://academic.oup.com/gigascience/pages/technical_note)
(the sentence regarding the RRID in the following section is then redundant and can be deleted).

We created a new bullet point below “licence” and deleted the sentence in the following section as requested.

Close
