# Supplementary material for: SeQuiLa-cov: A fast and scalable library for depth of coverage calculations
Source: Gigascience. 2019 Aug 5;8(8):giz094. doi: 10.1093/gigascience/giz094 (PMC6680061; doi:10.1093/gigascience/giz094)
Supplement: giz094_Response_to_Reviewer_Comments_Revision_2 [file giz094_response_to_reviewer_comments_revision_2.pdf]

Author's Response To Reviewer Comments

Close

July 9, 2019

The Editor of GigaScience

Dear Editor,

As requested, we added the following statement and citation in section: "Availability of supporting data and materials":

An archival copy of the code and supporting data is also available via the GigaScience database GigaDB [18].

Most sincerely
Tomasz Gambin, Ph. D.
ZSI-Bio group (http://biodatageeks.org)
Institute of Computer Science
Warsaw University of Technology
E-mail: tgambin@ii.pw.edu.pl

Close
