# Supplementary material for: SeQuiLa-cov: A fast and scalable library for depth of coverage calculations
Source: Gigascience. 2019 Aug 5;8(8):giz094. doi: 10.1093/gigascience/giz094 (PMC6680061; doi:10.1093/gigascience/giz094)
Supplement: giz094_Reviewer_1_Report_Original_Submission — Brent Pedersen -- 1/22/2019 Reviewed [file giz094_reviewer_1_report_original_submission.pdf]

Reviewer Report

Title: SeQuiLa-cov: A fast and scalable library for depth of coverage calculations

Version: Original Submission **Date:** 1/22/2019

Reviewer name: Brent Pedersen

Reviewer Comments to Author:

The authors present SeQuiLa-cov to enable fast coverage calculation within the SeQuiLa framework. The algorithm is described clearly and the software appears to have great scalability, especially in comparison to existing
solutions. I do have several points to address:
1. In the intro the authors motivate the need for fast coverage. One example is that it is used by CNV callers.
However, it's not clear to me that existing CNV callers could utilize the coverage information in SeQuiLa-cov as it's
in their table format. Would it require export? Can tools other than SeQuiLa use the full coverage information available?
How are the timings affected if export to a standard (BED) text format is required?
2. Since scalability and speed are a key focus of the paper, the authors should show how this scales on CRAM data.
Most centers and even small labs are moving to CRAM. In mosdepth, we show that we can get better speed with CRAM
especially in --fast-mode.
3. From the benchmarking scripts here:
http://biodatageeks.org/sequila/benchmarking/benchmarking.html it's not clear
to me how the window mode was run with mosdepth. Much of the time in mosdepth in spent writing the
per-base coverage which can be avoided if only the window information is needed by passing the `-n` flag.
4. This is related to 1. But, given the relative youth of SeQuiLa, it would be good to have a clear statement about how/if
SeQuiLa-cov results can be used outside of the SeQuiLa environment. If this is not feasible, that should be clear in the
text. If use of these fast coverage results does not require buy-in to the full SeQuiLa ecosystem, that would be a boon
for the software.
signed,
-Brent Pedersen

Level of Interest

Please indicate how interesting you found the manuscript: Choose an item.

Quality of Written English

Please indicate the quality of language in the manuscript: Choose an item.

Declaration of Competing Interests

Please complete a declaration of competing interests, considering the following questions:

- Have you in the past five years received reimbursements, fees, funding, or salary from an organisation that may in any way gain or lose financially from the publication of this manuscript, either now or in the future?
- Do you hold any stocks or shares in an organisation that may in any way gain or lose financially from the publication of this manuscript, either now or in the future?
- Do you hold or are you currently applying for any patents relating to the content of the manuscript?
- Have you received reimbursements, fees, funding, or salary from an organization that holds or has applied for patents relating to the content of the manuscript?
- Do you have any other financial competing interests?
- Do you have any non-financial competing interests in relation to this paper?

If you can answer no to all of the above, write 'I declare that I have no competing interests' below. If your reply is yes to any, please give details below.

I declare that I have no competing interests

I agree to the open peer review policy of the journal. I understand that my name will be included on my report to the authors and, if the manuscript is accepted for publication, my named report including any attachments I upload will be posted on the website along with the authors' responses. I agree for my report to be made available under an Open Access Creative Commons CC-BY license (http://creativecommons.org/licenses/by/4.0/). I understand that any comments which I do not wish to be included in my named report can be included as confidential comments to the editors, which will not be published.

I agree to the open peer review policy of the journal

To further support our reviewers, we have joined with Publons, where you can gain additional credit to further highlight your hard work (see: https://publons.com/journal/530/gigascience). On publication of this paper, your review will be automatically added to Publons, you can then choose whether or not to claim your Publons credit. I understand this statement.

Yes Choose an item.
