# Supplementary material for: SeQuiLa-cov: A fast and scalable library for depth of coverage calculations
Source: Gigascience. 2019 Aug 5;8(8):giz094. doi: 10.1093/gigascience/giz094 (PMC6680061; doi:10.1093/gigascience/giz094)
Supplement: giz094_Reviewer_3_Report_Original_Submission — Sara Goodwin -- 2/26/2019 Reviewed [file giz094_reviewer_3_report_original_submission.pdf]

Reviewer Report

Title: SeQuiLa-cov: A fast and scalable library for depth of coverage calculations

Version: Original Submission **Date:** 2/26/2019

Reviewer name: Sara Goodwin

Reviewer Comments to Author:

This manuscript covers a distributed computing method for calculating coverage from NGS data. The authors compare several commonly used methods of calculating coverage (GATK, samtools, bedtools etc.)
In general this manuscript explains the method well and show evidence that the SeQuiLa-cov tool has improved performance relative to the other tools it is compared too. There are two items that I feel would help the reader have a better understanding of the performance of the tool. 1: The authors compare samtools per-base result to the the SeqQuiLa-cov blocks and fixed length results, showing a significant reduction in time. This is reasonable given that samtools has not event or window level output. However, SeQuila-cov has a base level output in addition to event and window. It would be useful of the authors could also include this comparison. 2: Long-read sequencing is becoming a more important tool for WGS. Like with short read approaches, coverage is critical tool for a successful study. There has been very little work done on coverage calculator comparisons with long reads as a data input. It would be useful for the authors to discuss the performance of the tool on a long read data set.

Level of Interest

Please indicate how interesting you found the manuscript: Choose an item.

Quality of Written English

Please indicate the quality of language in the manuscript: Choose an item.

Declaration of Competing Interests

Please complete a declaration of competing interests, considering the following questions:

- Have you in the past five years received reimbursements, fees, funding, or salary from an organisation that may in any way gain or lose financially from the publication of this manuscript, either now or in the future?
- Do you hold any stocks or shares in an organisation that may in any way gain or lose financially from the publication of this manuscript, either now or in the future?
- Do you hold or are you currently applying for any patents relating to the content of the manuscript?

- Have you received reimbursements, fees, funding, or salary from an organization that holds or has applied for patents relating to the content of the manuscript?
- Do you have any other financial competing interests?
- Do you have any non-financial competing interests in relation to this paper?

If you can answer no to all of the above, write 'I declare that I have no competing interests' below. If your reply is yes to any, please give details below.

I declare that I have no competing interests

I agree to the open peer review policy of the journal. I understand that my name will be included on my report to the authors and, if the manuscript is accepted for publication, my named report including any attachments I upload will be posted on the website along with the authors' responses. I agree for my report to be made available under an Open Access Creative Commons CC-BY license (http://creativecommons.org/licenses/by/4.0/). I understand that any comments which I do not wish to be included in my named report can be included as confidential comments to the editors, which will not be published.

I agree to the open peer review policy of the journal

To further support our reviewers, we have joined with Publons, where you can gain additional credit to further highlight your hard work (see: https://publons.com/journal/530/gigascience). On publication of this paper, your review will be automatically added to Publons, you can then choose whether or not to claim your Publons credit. I understand this statement.

Yes Choose an item.
